# Elevated risk of adverse effects from foodborne contaminants and drugs in inflammatory bowel disease: a review

**DOI:** 10.1007/s00204-024-03844-w

**Published:** 2024-09-09

**Authors:** Tom Walraven, Mathias Busch, Jingxuan Wang, Joanne M. Donkers, Marjolijn Duijvestein, Evita van de Steeg, Nynke I. Kramer, Hans Bouwmeester

**Affiliations:** 1 Division of Toxicology, Wageningen University and Research, Wageningen, The Netherlands; 2 Department of Metabolic Health Research, Netherlands Organization for Applied Scientific Research (TNO), Leiden, The Netherlands; 3 Department of Gastroenterology and Hepatology, Radboud University Medical Center, Nijmegen, The Netherlands

**Keywords:** Inflammatory bowel disease, Chemically-induced disorders, Foodborne illnesses, Drug-related side effects and adverse reactions, Ulcerative colitis, Crohn’s disease

## Abstract

The global burden of Inflammatory bowel disease (IBD) has been rising over the last decades. IBD is an intestinal disorder with a complex and largely unknown etiology. The disease is characterized by a chronically inflamed gastrointestinal tract, with intermittent phases of exacerbation and remission. This compromised intestinal barrier can contribute to, enhance, or even enable the toxicity of drugs, food-borne chemicals and particulate matter. This review discusses whether the rising prevalence of IBD in our society warrants the consideration of IBD patients as a specific population group in toxicological safety assessment. Various in vivo, ex vivo and in vitro models are discussed that can simulate hallmarks of IBD and may be used to study the effects of prevalent intestinal inflammation on the hazards of these various toxicants. In conclusion, risk assessments based on healthy individuals may not sufficiently cover IBD patient safety and it is suggested to consider this susceptible subgroup of the population in future toxicological assessments.

## Introduction

The two major types of inflammatory bowel diseases (IBD), Crohn’s disease (CD) and ulcerative colitis (UC), are characterized by a chronically inflamed intestine. Between 1990 and 2017, the global number of cases of IBD rose from 3.7 million to 6.8 million, marking a strong increase in global prevalence of 85% (Alatab et al. 2020). While originally being labeled a “modern Western disease”, both the incidence and prevalence of IBD are now also rising in other parts of the world, as a Western lifestyle and diet are progressively being adopted in developing countries (Loftus 2004; Rizzello et al. 2019; Coward et al. 2022). The etiology of IBD remains unknown, but the complex interplay between genetic susceptibility, environmental risk factors, diet, and intestinal dysbiosis is thought to be of high importance (Leso et al. 2015). Genome-wide association studies identified a large number of IBD-associated susceptibility gene loci (Jostins et al. 2012). These include single nucleotide polymorphisms (SNPs) in receptor proteins linked to interactions with the intestinal microbiome (Ogura et al. 2001), proteins related to autophagy (Hampe et al. 2007), or interleukins (IL) and their receptors. Environmental factors that contribute to the pathogenesis of IBD include smoking (Bernstein et al. 2006), use of drugs (especially antibiotics; Shaw et al. 2010), stress (Bitton et al. 2008), emerging contaminants (Chen et al. 2023), ambient air pollution (Ananthakrishnan et al. 2011), and lastly, diet, both directly and indirectly by changing the intestinal microbiome (Wu et al. 2013; Knight-Sepulveda et al. 2015; Guo et al. 2024). Differences in the microbial composition have been found in IBD patients when compared to healthy individuals, although it is not clear whether this is a potential cause or a consequence of IBD (Joossens et al. 2011).

The disturbance of intestinal homeostasis, leading to the relapsing inflammation observed in IBD, is characterized by a variety of features on intestinal tissue- and cellular level. Although the initiating factors are poorly understood, studies found that IBD patients exhibit an impaired intestinal epithelial barrier (Maloy and Powrie 2011), as well as different mucin expression and secretion compared to healthy individuals (Furr et al. 2010; Sheng et al. 2011; Yamamoto-Furusho et al. 2015). This compromised barrier function results in heightened interaction and infiltration of toxicants and bacteria through the epithelium, triggering a reaction of the immune system (Johansson et al. 2014), resulting in an increased expression and release of pro-inflammatory cytokines (Shioya et al. 2007; Neurath 2014; Singh et al. 2016). For example, enterocytes can directly secrete IL-8 as a response to bacterial entry to attract macrophages (Eckmann et al. 1993). Phagocytosis and destruction of pathogens by macrophages leads to an immediate innate cellular immune response, characterized by the release of other cytokines like tumour necrosis factor-alpha (TNF-α; Pathmakanthan and Hawkey 2000; Jr et al. 2001). TNF-α is able to promote apoptosis and further dysfunction of the epithelial barrier (Van Antwerp et al. 1998; Wang et al. 2005, 2006), leading to a vicious cycle of continuous inflammatory responses. This chronic state of inflammation can induce oxidative DNA damage (Pereira et al. 2016), which ultimately increases the risk for cancer (Meira et al. 2008).

The most common clinical symptoms of IBD are diarrhea, abdominal pain, blood in stool and fatigue (Singh et al. 2011). Complications accompanying IBD include extra intestinal manifestations (Vavricka et al. 2015), intestinal fibrosis (Wang et al. 2022a, b), and an increased risk of developing colon cancer (Jess et al. 2012). Individuals suffering from IBD typically experience intermittent phases of exacerbation and remission (Zallot and Peyrin-Biroulet 2013), with specific stressors being identified for entering the next exacerbation phase (Singh et al. 2011). While CD can affect the entire gastrointestinal tract with alternating healthy and inflamed sites, UC is limited to the colon but shows a continuous area of inflammation (Yu and Rodriguez 2017). Despite some differences in typical symptoms and diagnostics, both CD and UC show a similar disease burden and generally share the same therapeutic strategies (e.g., suppressing inflammation; Le Berre et al. 2020). The long-term treatment target for IBD is endoscopically determined mucosal healing, the absence of disability and normalized health-related quality of life (as recently reviewed by the International Organization for the Study of Inflammatory Bowel Disease; Turner et al. 2021) Medical insights on effective therapies and side effects, evolve rapidly and are frequently discussed, for instance within the European Crohn’s and Colitis Organisation.

The prevalence of IBD is on the rise globally, raising concerns about the potential impact of environmental toxicants and drugs on affected individuals. While regulatory bodies conduct risk assessments of these compounds primarily on the general population, the specific effects on IBD patients remain largely unexplored. In this review, we discuss the potential risks that drugs, chemicals, and particles might pose to IBD patients, highlighting the need for greater attention to the unique vulnerabilities of IBD patients in these assessments.

## IBD from a toxicology perspective

### Foodborne chemical and particulate matter toxicity

**Fig. 1:**
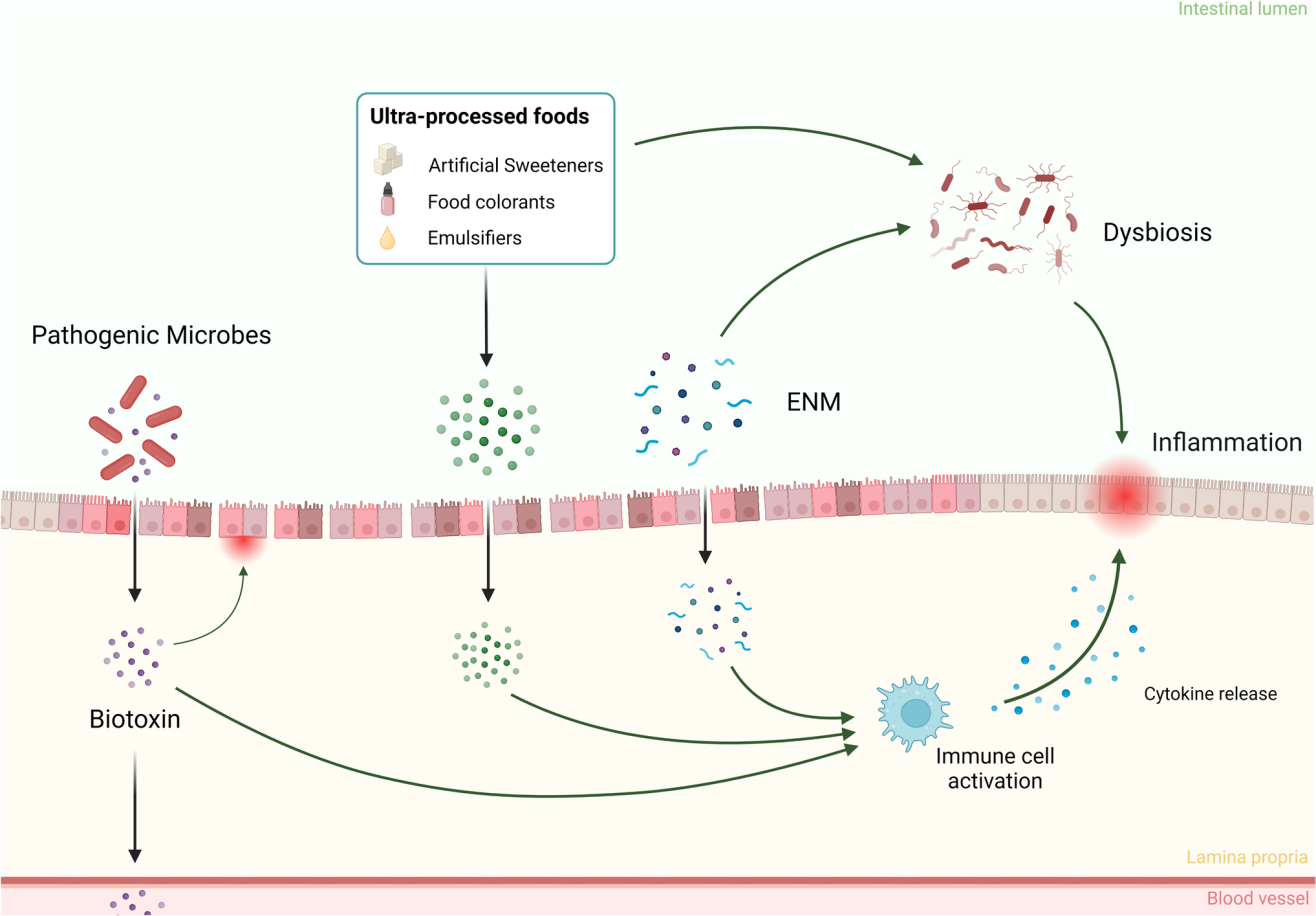
Dietary toxicity in IBD patients. Detrimental dietary additives, such as artificial sweeteners, emulsifiers and engineered nanomaterials (ENM) are able to enter the intestinal lamina propria as the epithelial barrier function is compromised, which can lead to activation of the immune system. Furthermore, these dietary compounds can disturb the microbiome, leading to further exacerbation of IBD. Biotoxins derived from pathogenic microbes such as *C. difficile* enter the lamina propria where they can further damage the intestine, as well as enter the systemic circulation. Created with BioRender.com

Dietary components are a primary environmental factor influencing gut health. In recent years, there has been a growing concern about the impact of foodborne contaminants and ultra-processed foods on the development and progression of intestinal diseases. Below, we discuss examples of foodborne contaminants, i.e., natural toxins, environmental contaminants, particulate matter, and chemicals that are deliberately introduced in food as additives (Fig. 1).

#### Foodborne biotoxins

Our environment is full of microorganisms that produce toxins which can have detrimental effects on gut (and systemic) health. As the gut barrier function is disrupted in IBD patients, toxins can be expected to more readily enter the body. Sera of patients with active CD or UC had higher levels of bacterial toxins from *Clostridium difficile*, *Escherichia coli O157*, *Salmonella Spp*., and *Staphylococcus aureus* compared to patients in remission (Qiu et al. 2014). Moreover, IBD patients are more susceptible to *C. difficile* infection, especially ulcerative colitis patients (Khanna et al. 2017). Furthermore, a retrospective study showed that prior antibiotic usage was associated with *C. difficile* toxin in stool samples of IBD patients (Meyer et al. 2004). The pathogenic mechanism of *C. difficile* is characterized by the production of two protein exotoxins (Toxin A and Toxin B) which compromise the epithelial barrier and induce inflammation (Hunt and Ballard 2013; Chandrasekaran and Lacy 2017). Not only does *C. difficile* infection lead to a worsening of IBD symptoms, it also increases adverse outcomes such as treatment failure, hospitalization, and even death (Sehgal et al. 2021). Other pathogenic and commensal bacterial species have shown similar opportunistic effects in IBD patients (Zhang et al. 2022). Some fungi are known to produce poisonous metabolites known as mycotoxins, which end up in our food. The most prevalent mycotoxin in our diet is deoxynivalenol (DON), produced by *Fusarium* species and commonly detected in cereals and other wheat-related products (Cano et al. 2013). DON is known to interfere with intestinal barrier function (Payros et al. 2020), and was found to exacerbate colitis in a DSS rodent model, even at otherwise no observed adverse effect levels (Gan et al. 2023). Furthermore, DON was found to disturb epithelial tight junctions by altering bile acid transport, and to increase proinflammatory cytokine production, in inflamed Caco-2/THP-1 co-cultures but not in control Caco-2 cultures (Wang et al. 2023). Other mycotoxins, such as aflatoxin and ochratoxin A have been identified as potential risk factors for IBD patients as well (Maresca and Fantini 2010).

#### Ultra-processed foods

A strong increase in the consumption of ultra-processed foods, such as fast food and frozen meals, can be seen throughout the world (Monteiro et al. 2013; da Costa et al. 2022). These highly-processed foods are typically energy dense, with high amounts of carbohydrates, fat, sugar, salt, and food additives (Monteiro et al. 2019). The increase in IBD prevalence in developing countries correlates with the increase of (ultra-)processed food consumption, prompting the question of what effect highly processed food has on gut health (Rizzello et al. 2019). Food additives like artificial sweeteners, such as aspartame, stevia, and sucralose, have been hypothesized to have a detrimental effect on gut health (Suez et al. 2015). Although artificial sweeteners are approved by the Food and Drug Administration (FDA) and European Food Safety Authority (EFSA), a multitude of epidemiologic and animal studies provide conflicting results on whether they induce intestinal dysbiosis and affect gut health in general (Ahmad et al. 2020; Raoul et al. 2022). Several studies found that sucralose exacerbates ileitis and colitis in different rodent models by inducing gut dysbiosis (Wang et al. 2019; Li et al. 2020; Guo et al. 2021). However, dietary levels of artificial sweeteners were only found to induce dysbiosis in animals with genetic predisposition for IBD, and not in healthy control mice (Rodriguez-Palacios et al. 2018), indicating that dietary levels of artificial sweeteners might only pose risks for IBD patients. Other major food components in ultra-processed foods are emulsifiers, which are used to stabilize food products by preventing separation of oils and water. Oral administration of low concentrations of the emulsifiers carboxymethylcellulose (CMC) and polysorbate-80 (P80) induced severe colitis in IL-10 knockout mice, but only mild inflammation in wild-type mice (Chassaing et al. 2015). Furthermore, exposure to the emulsifier carrageenan led to the aggravation of colitis in multiple rodent models (Bancil et al. 2021). Similar results have been found for other emulsifiers and thickeners, such as maltodextrin (Laudisi et al. 2019) and methylcellulose (Llewellyn et al. 2018). Synthetic food colorants are widely used in dietary products, especially in ultra-processed food. The common food colorants azo dye Red 40 and Yellow 6 are deemed safe for consumption at reported use levels by industry (Barciela et al. 2023). However, these azo dyes are metabolized by commensal bacteria into 1-amino-2-naphthol-6-sulfonate sodium salt, which was shown to exacerbate colitis in susceptible mice by promoting 5-hydroxytryptamine (5-HT) secretion, consequently leading to increased inflammation (He et al. 2021; Kwon et al. 2022), an observation that needs to be confirmed in humans.

#### Foodborne micro- and nanoparticles

Many processed foods contain engineered nanomaterials (ENM) purposely added as coloring agents, anticaking agents or as preservatives (de Oliveira et al. 2022). The total human uptake of ENM such as titanium dioxide (TiO_2_) or silica (SiO_2_) is challenging to quantify, but daily exposures are estimated to be up to 10.4 mg kg^−1^ body weight (bw) per day and 1.8 mg kg^−1^ bw per day (Dekkers et al. 2011; EFSA 2016), respectively. The safety of ENMs as additives is a highly debated topic (EFSA et al. 2021), as both TiO_2_ and SiO_2_ nanomaterials can induce pro-inflammatory reactions via the NOD-, LRR- and pyrin domain-containing protein 3 (NLRP3) inflammasome pathway in vitro (Busch et al. 2022a, b; Bredeck et al. 2023). This pathway is part of the innate immune system and is crucially involved in intestinal inflammation (Busch et al. 2022) and the pathogenesis of IBD (Bauer et al. 2012; Zhen and Zhang 2019). Although only minor effects were observed after oral exposure to nanoparticles in vivo (Wang et al. 2007; Chen et al. 2017), the outcome of numerous other studies spurred the hypothesis that oral exposure to particulate matter can have a negative impact on an already existing intestinal inflammation, such as in active IBD, instead of inducing it. In 2001, Lomer and colleagues observed a significantly reduced Crohn’s disease activity index (CDAI) in patients on a specific diet low on microparticles such as TiO_2_ (Lomer et al. 2001). However, the outcomes of this small pilot study (10 subjects per group) could not be confirmed in a larger follow-up study (91 subjects per group; Lomer et al. 2004). Nevertheless, in vivo studies in colitis mouse models also suggest that the intake of micro- and nanoparticles like polystyrene or TiO_2_ can exacerbate pre-existing intestinal inflammation (Ruiz et al. 2017; Zheng et al. 2021; Wang et al. 2022a, b). Similar observations were made in Caco-2, mucus secreting HT29-MTX-E12 cells and THP-1 derived macrophage based in vitro models of intestinal inflammation, where microplastics or metallic ENM caused effects only or more pronounced in the inflamed-like state of the model (Kämpfer et al. 2020; Busch et al. 2021).

Recently, micro- and nanoplastics have emerged as a contaminant of concern in food and drinking water that might impact gut health (Vethaak and Legler 2021; Niu et al. 2023; Busch et al. 2023). PET microplastics have shown to affect human gut microbiome compositions (Tamargo et al. 2022), and exposure to nano- and microplastics may affect intestinal functions such as intestinal epithelial permeability (Hirt and Body-Malapel 2020). While there have been some reports on the risk of plastic particles for IBD patients, our knowledge is still limited (Yan et al. 2022; Zhao et al. 2023; Zolotova et al. 2023). The ubiquitous nature of micro- and nanoplastics in our food chain warrants more investigations on the implications of these particles on the possibly attenuated risks for IBD patients.

#### Residues of agrochemicals

**Fig. 2:**
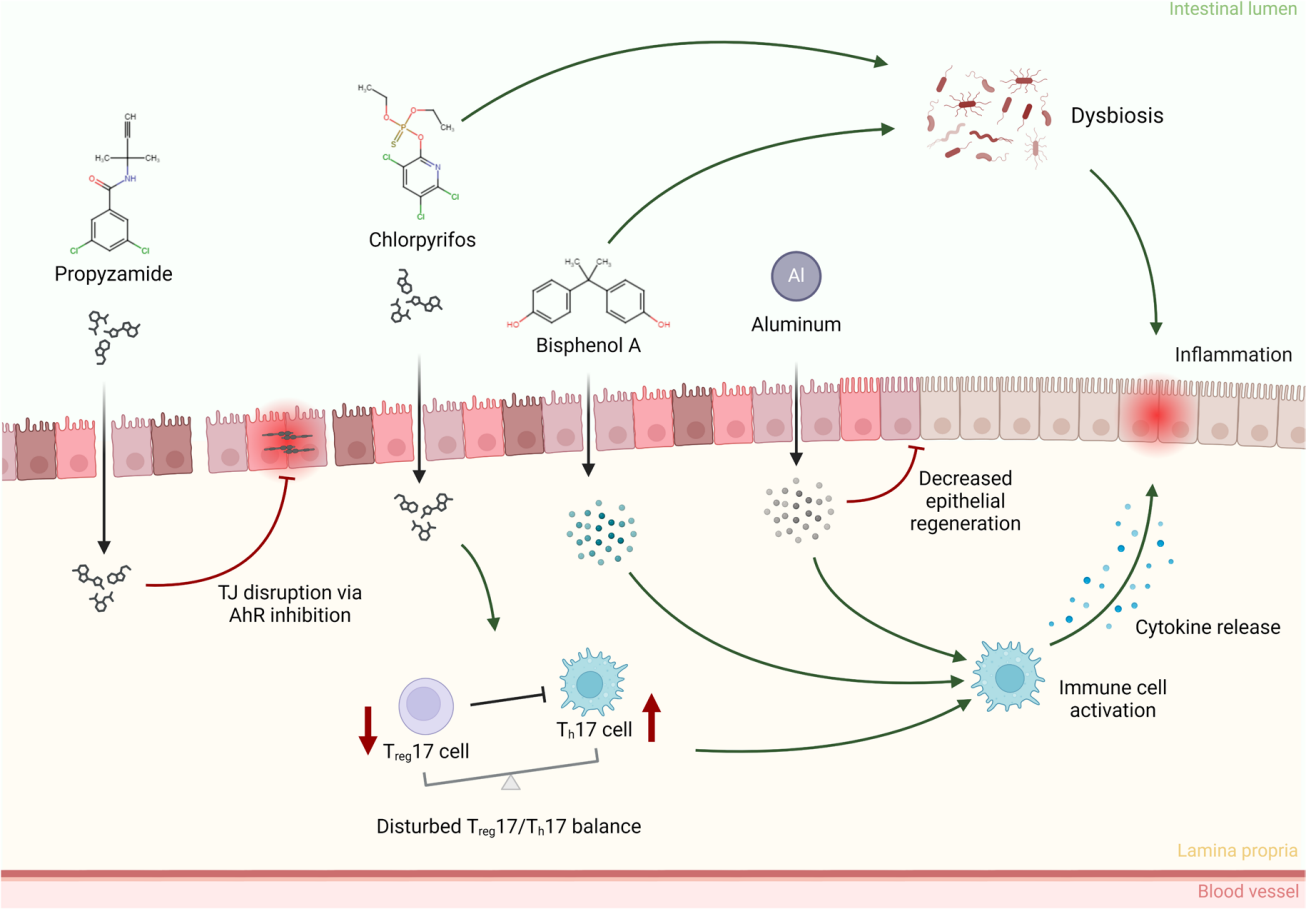
Chemical toxicity in IBD patients. Various chemicals have proven to have a deleterious effect on gut health of individuals with IBD. For example, Propyzamide inhibits AhR signaling leading to tight junction (TJ) disruption. The pesticide chlorpyrifos has been found to alter gut microbiome composition and to disturb T_reg_17/T_h_17 balance, resulting in increased inflammation. Endocrine-disrupting chemicals such as bisphenol A have been found to aggravate IBD. Aluminium has shown to worsen colitis, and decrease epithelial regeneration in mice. Created with BioRender.com

Chemical herbicides and pesticides are widely used in agriculture to prevent the growth of weeds and to protect our crops from pests to ensure the availability of food (Fig. 2) (Sharma et al. 2019). However, exposure to residues of these agrochemicals can influence IBD development and progression, as has recently been shown in an epidemiologic study correlating organochlorine exposure with an increase in incidence of IBD (Chen et al. 2024). This confirmed earlier observations in rodents. For example, the herbicide propyzamide has been found to increase inflammation and immune cell infiltration in mice models for colitis and enteritis by inhibiting AhR nuclear receptor mediated signaling, which was not observed in healthy mice (Sanmarco et al. 2022). While the use of organophosphate pesticides like chlorpyrifos (CPF) has been banned in the EU (EFSA 2019), humans are still exposed to residues of these pesticides because of its intensive use in the past decades (Hongsibsong et al. 2020; Foong et al. 2020; EFSA 2023). A limited number of studies showed the detrimental effects of CPF on the gut. CPF was found to disturb the balance between T_reg_17 and T_h_17 cells in a DSS-induced colitis mouse model, leading to further aggravation of tissue injury (Huang et al. 2019, 2020). In the healthy mammalian gut, Th17 cells protect the host by secreting proinflammatory cytokines, while Treg cells restrain excessive effector T-cell responses (Lee 2018). However, in IBD patients this T_reg_ 7/ T_h_17 balance is disturbed, resulting in inflammation (Yan et al. 2020). Additionally, CPF was found to alter the gut microbiota composition in mice, which led to an increase in intestinal inflammation and permeability (Zhao et al. 2016). Other active ingredients in chemical herbicides such as dicamba (Mesnage et al. 2021) and 2,4-Dichlorophenoxy acetic acid (Tu et al. 2019) have shown detrimental effects on gut homeostasis as well. Importantly, long-term intestinal effects upon human exposure to (residues of) pesticides are currently not incorporated in the evaluation for market authorization of agrochemicals (Gangemi et al. 2016).

#### Metal residues in food

Industrialization has led to an accumulation of metals, particularly aluminum, in our food and drinking water (Alasfar and Isaifan 2021). Oral administration of aluminum at levels comparable to high daily intake by humans in urban regions (1.5 mg kg^−1^ day^−1^) aggravated inflammation in three different mouse models of colitis, evidenced by increased proinflammatory cytokine production, heightened macroscopic and histological inflammation, and decreased epithelial regeneration (Pineton de Chambrun et al. 2014). In a follow-up study using human tissues, aluminum induced cytokine secretion in colon tissue isolate from CD patients but not in tissue from healthy individuals (Djouina et al. 2022). The role of other metals in IBD have been described as well, but only in a handful of studies. Nickel particles were found to be aggregated in nickel sites (Ø10–100 µm) in intestinal tissue of CD patients, and where found to exacerbate colitis in a DSS mouse model and induced colitis in mice genetically susceptible to inflammation (Matsuda et al. 2022). Low dietary levels of manganese seem to exacerbate colitis in DSS mice (Choi et al. 2020; Paschall et al. 2020), and arsenic is known to cause intestinal barrier disruption in vitro using intestinal Caco-2 cells (Chiocchetti et al. 2019). The high concentration of metals in our environment require more in-depth research on their potential toxicity in both healthy individuals and susceptible individuals.

#### Endocrine disruptors in food

Endocrine-disrupting chemicals are exogenous chemicals that interfere with hormonal processes such as growth, development, reproduction and metabolism. Endocrine disrupting chemicals are mostly by-products of the industrial manufacturing and use of plastics, pesticides, pharmaceuticals, and flame-retardants (Benotti et al. 2009; Schug et al. 2011). Bisphenol A (BPA) is a representative chemical of a large class of chemical compounds that are widely used in the production process of plastics, although the use of BPA is currently being restricted (EFSA Panel on Food Contact Materials, Enzymes and Processing Aids (CEP) et al. 2023). An observational study in CD patients found that patients with high serum levels of BPA had an increased systemic inflammatory response (Linares et al. 2021). Endocrine receptor levels were significantly increased and correlated with BPA levels. Furthermore, markers for microbial dysbiosis such as bacterial DNA and endotoxin levels in the blood were correlated with increased BPA uptake. A metagenomic analysis in mice revealed that dietary BPA intake reduces the species diversity of the intestinal microbiome (Lai et al. 2016). Loss of microbial diversity is associated with a multitude of chronic illnesses, including IBD (Flight et al. 2015; Gong et al. 2016; Wilkins et al. 2019). Accordingly, BPA was found to alter microbiome-related metabolite levels, and thereby aggravating disease activity, in DSS-colitis models (DeLuca et al. 2018). Both BPA and its substitute fluorene-9-bisphenol were also found to deregulate sugar and fatty acid metabolism in colitic mice (Yin et al. 2022). The reduced human health based guidance values for BPA, (EFSA Panel on Food Contact Materials, Enzymes and Processing Aids (CEP) et al. 2023) could potentially result in the rise of other bisphenols, which, although untested, might also pose toxic effects.

#### Conclusions on potential increased risk of IBD patients upon exposure to foodborne chemicals

Humans are exposed to a great diversity of chemicals via food and drinking water. A distinction in two groups of chemicals can be made. First, chemicals that require a marketing authorisation (i.e., agrochemicals and food additives) and therefore undergo a regulatory safety assessment before use is permitted. While these chemicals are extensively evaluated, the development of intestinal inflammation or IBD is not considered in toxicological safety testing programmes. Secondly, chemicals can end-up in our food as contaminants via the environment. While for some of these chemicals limits for their tolerated presence in food are in place, the true effects on susceptible groups in the population remain largely unknown. Together, this emphasizes the need for adequate testing approaches to study the mode of action of chemicals and their potential relation to IBD, or to assess the potential increased vulnerability of IBD patients upon exposure to foodborne chemicals.

### Potential increased risk of IBD patients upon exposure to drugs

**Fig. 3:**
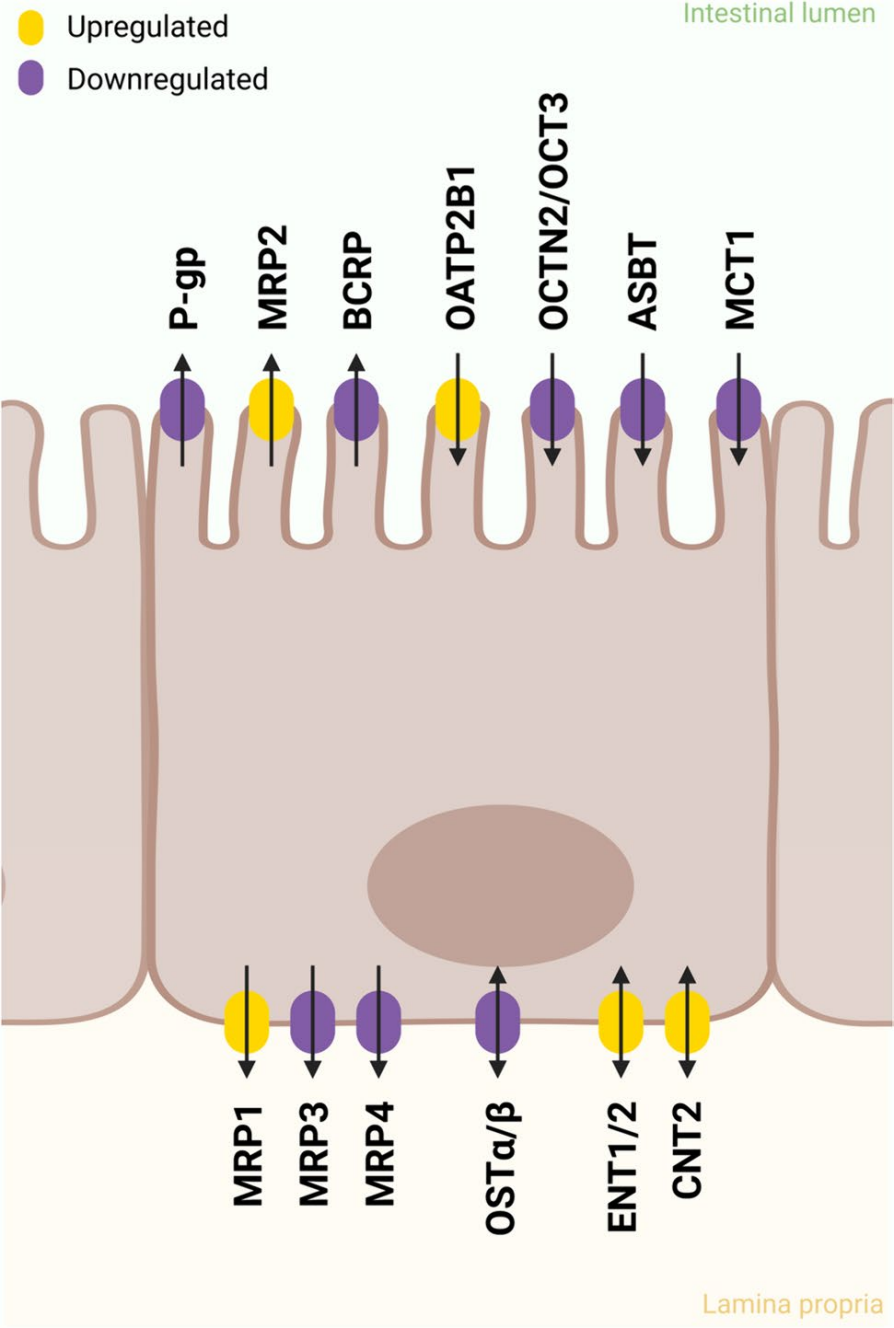
Altered transporter expression levels in IBD patients, divided into apical and basolateral transport. Upregulated transporters are shown in yellow, while downregulated transporters are shown in purple. Created with BioRender.com

**Fig. 4:**
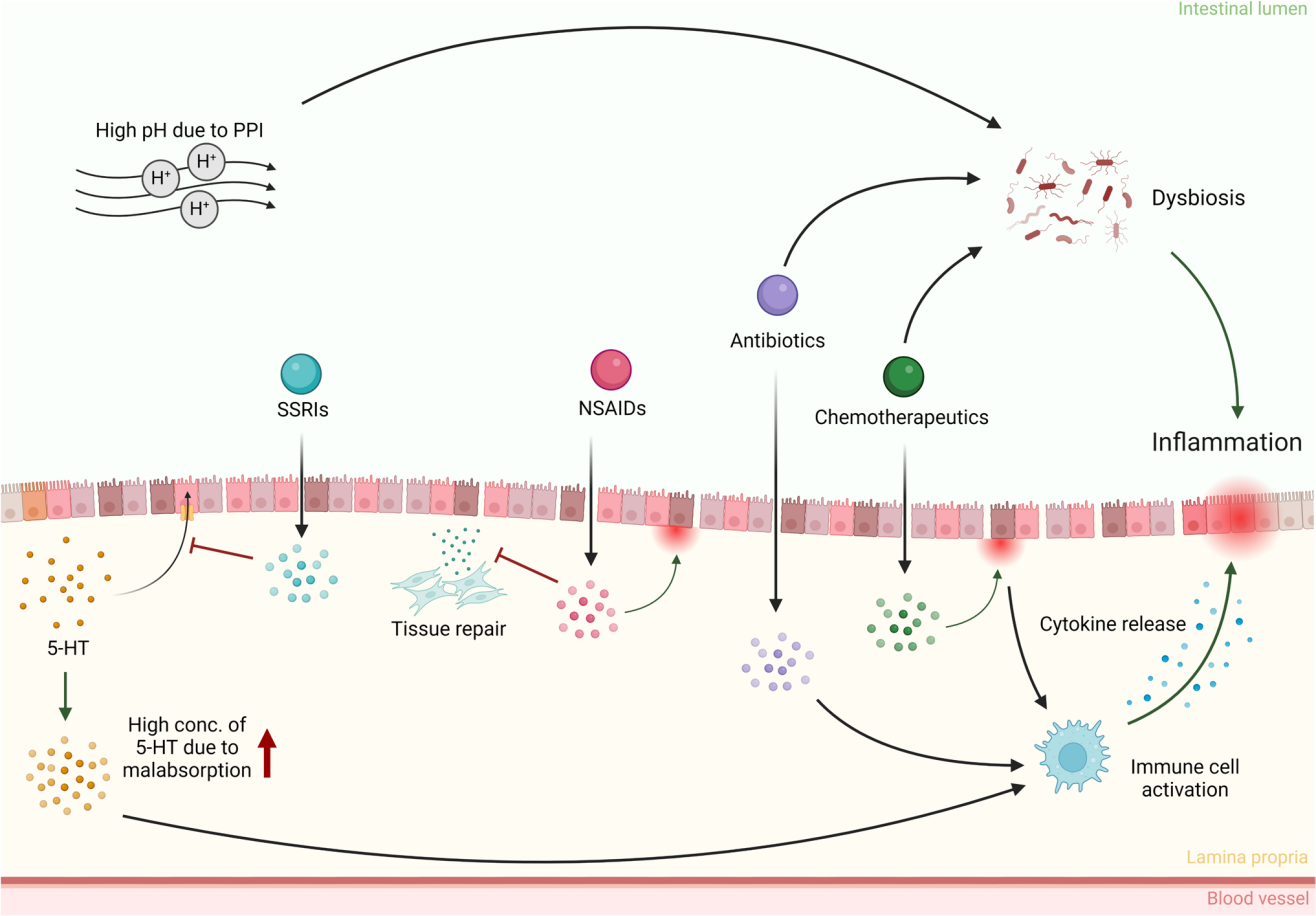
Toxicity induced by pharmaceuticals in IBD patients. The compromised epithelial barrier in IBD patients results in increased uptake of compounds. Chemotherapeutics are cytotoxic to the intestinal epithelium, releasing damage associated proteins leading to increased inflammation. Nonsteroidal anti-inflammatory drugs (NSAIDs) damage the intestinal epithelium by disrupting oxidative phosphorylation, and prevent tissue repair via COX inhibition. Proton pump inhibitors increase the luminal pH, leading to gut microbial dysbiosis. Antibiotics cause dysregulation of both the immune response and the gut microbiome. Selective serotonin reuptake inhibitors (SSRIs), inhibit serotonin (5-HT) reuptake by enterocytes by blocking selective serotonin reuptake inhibitors. The increased 5-HT concentration in the lamina propria results in immune cell activation, which leads to increased intestinal inflammation. Created with BioRender.com

Recent studies have shown that some drugs (i.e., drugs not related to IBD therapy) may pose a higher health risk for IBD patients compared to healthy individuals. These heightened risks can be attributed to several factors. First, as the intestinal epithelium of IBD patients in the active phase of IBD is characterized by increased crypt apoptosis and villus atrophy, leaving the mucosal tissue open to luminal contents (Sonis 2004), drugs might further induce epithelial damage and increase intestinal permeability. This allows the translocation of other harmful substances and pathogens across the epithelial barrier, possibly exacerbating the inflammatory state and worsening IBD symptoms. Secondly, alterations in the presence or activity of enterocyte transporters in IBD patients can affect drug pharmacokinetics, impacting the efficacy and increasing the risk of adverse effects of the drugs (Yoshida et al. 2013). It has been reported that the mRNA expression of equilibrative nucleoside transporter (*ENT*) 1/2, concentrative nucleoside transporter (*CNT*) 2, organic anion-transporting polypeptide (*OATP*) 2B1 (Wojtal et al. 2009), and protein levels of multidrug resistance protein (MRP) 1 and MRP2 were significantly elevated (Ufer et al. 2009; Erdmann et al. 2019), whereas the protein levels for apical sodium-dependent bile acid transporter (ASBT), organic solute transporter (OST), novel organic cation transporter (OCTN) 2 (Erdmann et al. 2019), OCT3, monocarboxylate transporter (MCT) 1, P-glycoprotein (P-gp), breast cancer resistance protein (BCRP; Ufer et al. 2009), MRP3 (Jahnel et al. 2014), and MRP4 (Verma et al. 2013) were significantly lower in the intestine of IBD patients (Fig. 3). In the following paragraphs we discuss different classes of drugs that can either cause additional intestinal toxicity or have increased bioavailability due to increased transporter activity in IBD patients (Fig. 4).

#### Proton pump inhibitors

Drugs such as omeprazole, esomeprazole and lansoprazole, are commonly applied proton pump inhibitors (PPIs) used for the treatment of gastroesophageal disorders (Khan and Howden 2018). A recent human study revealed a correlation between the administration of PPIs to IBD patients and an elevated risk of their hospitalization (Nighot et al. 2023). One of the causes is thought to be the disruption of intestinal tight junctions by the PPI-induced increase in extracellular pH levels, triggering the activation of myosin light chain kinase via p38 pathways, as shown in DSS-induced colitis mouse models (Nighot et al. 2023). Secondly, PPIs increase gastric pH levels at standard therapeutic doses, allowing harmful bacteria to survive the gastric passage, which could result in alterations of the intestinal microbial composition (Lombardo et al. 2010). Both modes of action are proposed to underly PPI-induced exacerbation of IBD symptoms.

#### Nonsteroidal anti-inflammatory drugs

Nonsteroidal anti-inflammatory drugs (NSAIDs), including ibuprofen and naproxen, are in general extensively prescribed due to their effectiveness in the treatment of inflammation and pain (Mahadevan et al. 2002). Usage of NSAIDs is associated with an elevated risk of intestinal mucosal damage and related complications. Despite having anti-inflammatory properties, several studies have reported exacerbation and relapses in IBD patients upon NSAIDs administration (Kaufmann and Taubin 1987; Felder et al. 2000; Forrest et al. 2004). NSAIDs exhibit their anti-inflammatory and analgetic effects primarily by inhibiting the activity of cyclooxygenase (COX). COX is also responsible for the production of prostaglandins involved in tissue repair and ulcer healing processes in IBD patients (Halter et al. 2001) indicated by increased COX gene expression in the inflamed colon (Lin et al. 2018). As a consequence, the inhibition of COX and prostaglandin production by NSAIDs compromises the recovery of the intestinal barrier and further increases intestinal permeability in IBD patients (O’brien 2000). In addition, at the moment of intestinal absorption, NSAIDs cause specific damage to enterocyte mitochondria by disrupting oxidative phosphorylation, resulting in enterocyte cytotoxicity and further increased intestinal permeability (Matsui et al. 2011). The chronic inflammation and ulceration present in IBD patients weakens the intestinal lining, rendering it more susceptible to NSAIDs-induced barrier damage and increasing the risk of (further) ulceration, perforation, and bleeding.

#### Antibiotics

Studies have shown that the gut microbiome is altered in the intestine of IBD patients, prompting the use of antibiotics as a therapeutic strategy (Nitzan et al. 2016). Interestingly, several cohort studies have shown that there is an association between early-life antibiotic exposure and the development of IBD (Margolis et al. 2010; Hviid et al. 2011; Lee et al. 2013). Clinicians are advised to be cautious when prescribing antibiotics to IBD patients (Theochari et al. 2018). The compromised intestinal barrier in IBD patients results in an increased penetration of antibiotics across the epithelium into the lamina propria, where the intestinal immune cells are located. Studies have shown that therapeutic levels of the antibiotic drugs gentamicin and amikacin reduced the chemotaxis of polymorphonucleocyte (PMN), which are recruited from blood vessels in response to inflammation in IBD patients (Goodhart 1977; Khan et al. 1979). This might cause dysregulated immune responses and increased inflammation. In addition, antibiotics disrupt the composition of gut microbiome and decrease microbial diversity, providing pathogenic microbes with the opportunity to overgrow the intestine (Yoon and Yoon 2018), further driving the vicious cycle that is IBD.

#### Selective serotonin reuptake inhibitors

Apart from the direct intestinal clinical adverse outcomes, individuals suffering from IBD are also at increased risk of developing depression due to a significant drop in life quality (Geiss et al. 2018). In general, selective serotonin reuptake inhibitors (SSRIs) are used as antidepressant medications, and are primarily acting by inhibiting the reuptake of the neurotransmitter 5-HT, better known as serotonin, thereby increasing neuroactivity in the brain (Jones and Blackburn 2002). Specifically for IBD patients, the use of SSRIs might cause concern. To prevent prolonged receptor stimulation, 5-HT is actively taken up by the serotonin transporter (SERT), which is distributed throughout the intestinal epithelium (Coates et al. 2017). In IBD patients, there is reduced expression of SERT, leading to decreased uptake of 5-HT and consequently elevated levels of 5-HT in the *lamina propria* (Coates et al. 2004). The inhibition of SERT activity by SSRIs administration further increases the concentration of 5-HT levels in the *lamina propria*, which then activate immune cells such as macrophages and mast cells, promoting the production of pro-inflammatory cytokines (Shajib and Khan 2015). Mice studies showed that knockout of SERT exacerbates colitis and intestinal inflammation in IL-10 deficient mice (Bischoff et al. 2009; Haub et al. 2010). In addition, the potentiation of serotonergic signaling in SERT knockout mice contributes to watery diarrhea, which is one of the symptoms of IBD patients (Haub et al. 2010). Although human studies are lacking, this suggests an increased risk of complications for IBD patients using SSRIs.

#### Chemotherapeutic drugs

IBD has been associated with higher incidences of malignancies, such as colon cancer due to chronic inflammation, or lymphomas and non-melanoma skin cancers due to prolonged use of IBD therapeutic drugs (Laredo et al. 2023). Vice versa, anticancer treatments, such as the chemotherapeutic drug 5-fluorouracil, have been reported to exacerbate diarrhea in IBD patients, most likely by inducing mitotic arrest and apoptosis of crypt cells leading to altered fluid transport (Stein et al. 2010; Shawna Kraft 2013). As stated above, the altered expression and activity of transporters in IBD patients can change the pharmacokinetics of orally applied drugs. This is exemplified by drugs that are a substrate for the apical efflux transporter P-gp, which expels substrate drugs from epithelial cells to the lumen of the intestine and thereby limits the absorption of drugs (Estudante et al. 2013). Inhibited P-gp activity has been found to significantly increase the area under the plasma concentration–time curve (AUC) and the plasma peak concentration (C_max_) of chemotherapeutics like paclitaxel (Meerum Terwogt et al. 1999), topotecan (Kuppens et al. 2007), and doxorubicin (Planting et al. 2005) in humans upon oral administration, potentially increasing the magnitude of their adverse effects due to higher blood and tissue concentrations.

#### Conclusions on potential increased risk of IBD patients upon exposure to drugs

The intestinal epithelium of IBD patients has an altered activity of enterocyte transporters and has a lower barrier function compared to healthy individuals. Therefore, the pharmacokinetics and the local toxicodynamics of drugs might be different in IBD intestinal cells, for instance because of altered drug receptor expression. Indirect harmful effects that drugs might have on the intestinal microbiome also need to be considered when investigating the risks of these compounds.

## Models for inflammatory bowel diseases

In this section, we summarize experimental models of IBD and discuss their advantages and disadvantages in toxicity testing of possible inflammation-exacerbating toxicants. For in-depth discussions on the use of models to investigate the pathogenesis, pathophysiology and treatment of IBD, we refer the reader to more detailed reviews on these topics (Dieleman et al. 1997; Cominelli et al. 2017; Joshi et al. 2022).

### Animal models of IBD

Experimental animal models have proven to be valuable tools in understanding the basic pathophysiology of IBD, however such models can also be used to study the effects of chemical exposure on the progression of IBD. The first experimental model for colitis was developed in 1957 by sensitizing rabbits to crystalline egg albumin by rectal administration of diluted formalin (Kirsner and Elchlepp 1957). Ever since, various animal models have been developed to investigate IBD, most of which are rodents expressing acute or chronic colitis. Here, we describe three categories of commonly used rodent models and their potential (and limitations) for toxicity testing (Table 1).

**Table 1:** Rodent models for IBD

Mouse model	Main mode of onset	IBD form	Disease onset	Histopathological presentation	Pros	Cons	Example study
DSS	Disruption of epithelial barrier	UC	3–7 days	Mucosal ulceration, severely disturbed tissue architecture, edema, (sub)mucosal inflammation, immune cell infiltration	Low costs, easy administration, rapid disease onset, dose-controllable disease severity	Non-specific injury, non-immune-mediated inflammation, acute inflammation	(Ruiz et al. 2017)
TNBS	Activation of intestinal immune response	CD	3–7 days	Mucosal ulceration, severely disturbed tissue architecture, edema, transmural inflammation, immune cell infiltration, fibrosis (chronic), dysplasia (chronic)	Low costs, easy administration, fast disease onset, immune-mediated inflammation, both acute and chronic inflammation possible	Nonspecific injury, low clinical relevance	(Amamou et al. 2021)
IL-10 knockout	Th1/Th17 dysregulation	UC	3 months	Mucosal ulceration, chronic (sub)mucosal inflammation, crypt abnormalities, immune cell infiltration, fibrosis, dysplasia	Immune-mediated inflammation, clinically-relevant gene	Late disease onset, low severity of disease, interlaboratory variability, costly	(Wilson et al. 2011)
T-cell transfer	Immune dys-regulation	Pancolitis	5–8 weeks	Transmural inflammation, edema, crypt abnormalities, immune cell infiltration, dysplasia	Immune-mediated inflammation, synchronized disease onset, suitable for immunologic studies, high reproducibility	Laborious process, costly, interlaboratory variability, spontaneous formation of T-cells, nonspecific to CD/UC	(Fort et al. 2001)
SAMP1/YitFC	Spontaneous	CD	10 weeks	Focal lesions of: Mucosal ulceration, edema, transmural inflammation, crypt abnormalities, immune cell infiltration	High clinical relevance to CD, suitable for immunologic studies, chronic inflammation	Late disease onset, costly, interindividual variation	(Rodriguez-Palacios et al. 2018)

One of the main categories of animal models for IBD are the chemically-induced rodent models. Oral administration of dextran sulfate sodium (DSS) to mice and rats leads to self-limiting, acute inflammation that resembles UC. DSS disrupts the gut barrier function by inducing direct damage to the epithelium, allowing infiltration of luminal antigens into the *lamina propria* (Chassaing et al. 2014). Trinitrobenzene sulfonic acid (TNBS) in combination with ethanol induces bowel inflammation reminiscent of CD when administered rectally to rodents by inducing an immune response (Antoniou et al. 2016). However, chemically-induced rodent models have important limitations, as they induce a nonspecific injury to the intestinal epithelium. Furthermore, animal strain, gender, and whether these animals are germ-free will affect disease susceptibility (Koboziev et al. 2011), and concerns have been raised on the high severity of the induced disease and consequently the susceptibility to toxicants.

Transgenic rodent models are the second main type of IBD animal models. A widely used transgenic model in IBD research is the *IL-10* knockout mouse. The immunoregulatory cytokine IL-10 maintains intestinal immune homeostasis mainly via T-helper (Th)1 and Th17 cells (Jacobse et al. 2021). Inhibition of IL-10 results in excessive secretion of proinflammatory cytokines (Gunasekera et al. 2020). In *IL-10* knockout mice, this has been shown to result in colitis (Keubler et al. 2015). Interestingly, germ-free *IL-10* knockout mice do not develop spontaneous colitis (Sellon et al. 1998), suggesting a crucial role of external pathogens in the onset of colitis. Secondly, as T-lymphocytes are key mediators of chronic inflammation in the gut, transgenic CD4^+^CD45^high^ T-cell mice models have been developed (Ostanin et al. 2009). A large advantage of this model is that it can be used to investigate the effect of toxicants on early-stage immunologic events associated with IBD. A drawback of both of these immunomodulatory models is that the microbiome composition of mice differ between research facilities which can cause differences in colitis development and in susceptibility of animals to toxicants (Reinoso Webb et al. 2018; Ericsson and Franklin 2021).

Mouse models spontaneously developing intestinal inflammation can be considered the third type of IBD models. The SAMP1/YitFc mouse strain develops a CD-like phenotype without chemical, genetic, or immunogenic manipulation that closely resembles CD in humans (Kosiewicz et al. 2001). These mice show lesion formation in the terminal ileum paired with a discontinuous pattern of normal mucosa and inflammed mucosa. Most of the mice develop this chronic ileitis at the age of 10 weeks (Rivera-Nieves et al. 2003). Due to its close resemblance to chronic CD in humans, the model can provide important insights in the inductive and exacerbating effects of xenobiotics on IBD. However, the long duration needed for complete disease onset makes it an expensive and time-consuming model to use.

Although frequently used in preclinical IBD research, animal models show uncertainty regarding their accuracy in predicting the human physiological response to drugs, chemicals and other toxicants (Leenaars et al. 2019). In addition, rising costs of animal studies, ethical concerns, and high drug attrition rates have enticed researchers to develop more advanced in vitro models in an attempt to reduce, or replace, animal testing, as well as to enable high-throughput testing of toxicants.

### Ex vivo* and *in vitro* models for IBD*

Several types of intestinal epithelial and intestinal tissue ex vivo and in vitro models have been developed for toxicokinetic and toxicodynamic studies. These models have turned out to be powerful models to study the molecular and cellular processes underlying the pathophysiology of IBD, and to study the interactions with drug and foodborne chemicals as discussed above. Here, we review the current state of the art of these models and discuss further outlooks for the use of ex vivo and in vitro models.

#### Ex vivo* models*

Intestinal tissue explants resemble the in vivo architecture and cell type diversity and are therefore highly relevant to understand the impact of nutrients, drugs, and toxicants in a physiologic setting (Donkers et al. 2021; Rahman et al. 2021). IBD patient-derived material maintains disease characteristics like the impaired intestinal barrier, local inflammation, and intestinal fibrosis which therefore do not need to be induced artificially. Consequently, the impact of the IBD-phenotype on intestinal processes like drug or toxicant absorption can be studied in a representative model. However, the use of ex vivo gut tissue for IBD research is still limited, mostly through the constrained throughput and lifespan (hours to a maximum of 3 days) of these tissue explant models, but important steps have been taken over the recent years.

Two well-known ex vivo gut tissue model are the Ussing chamber and InTESTine™ model (Westerhout et al. 2014; Stevens et al. 2019). In these models, tissue segments are clamped vertically (Ussing chamber) or horizontally (InTESTine™) between two chambers, allowing the measurement of transport across the epithelial barrier (Westerhout et al. 2014; Stevens et al. 2019). These devices are mainly used to study drug absorption or gut tissue barrier functions. Clamping intestinal tissue of UC patients (Nakai et al. 2020) and CD patients (Biskou et al. 2022) demonstrated a leaky barrier mainly for the paracellular passage route. Furthermore, barrier permeability was increased in inflamed sites compared to non-inflamed sites (Libertucci et al. 2018), and remained leakier than normal (compared to IBD patients and healthy controls) during disease remission (Katinios et al. 2020). Ex vivo intestinal tissue explants were used to evaluate effectiveness of the TNF-α neutralizing antibody–drug Infliximab (Yakymenko et al. 2018). So far, impaired barrier function remains the only IBD-characteristic studied ex vivo. Insights into inflammation, disturbed processes in the supportive connective tissue, or altered absorption for specific drugs and toxicant, remain to be explored in tissue explant models.

#### Immortalized cell line (co-)culture models

The most widely used cell model exploits immortalized human colorectal adenocarcinoma cells (Caco-2 cells) that spontaneously differentiate upon reaching confluence into an adherent monolayer that shows features of enterocytes in the small intestine (Lea 2015). A wide range of compounds have been utilized to induce an inflammatory phenotype in the Caco-2 model (Table 2). The endotoxin lipopolysaccharide (LPS) is used to induce inflammation via the Toll-like receptor 4 (TLR4) pathway (Lu et al. 2008; Wang et al. 2023). Recombinant proinflammatory cytokines such as TNF-α and IL-1β induce a disease-like state in the Caco-2 model (Maria-Ferreira et al. 2018; Liang et al. 2020). Similar to some animal models, chemicals have been employed to induce damage in Caco-2 models, however they are not frequently used due to their non-representative nature to human inflammation (Araki et al. 2006; Toutounji et al. 2020). Lastly, intestinal epithelial injury can be induced via hypoxic or heat stress as well (Lian et al. 2021). On the downside, Caco-2 cells demonstrate an increased expression of crucial transporter proteins, including P-gp, MRP1, and OATP2B1, with levels ranging from 3- to 130-fold higher than those found in human jejunal tissue (Vaessen et al. 2017). As this does not reflect the expression of transporters in exacerbated phase IBD patients (see section above), employing alternative in vitro models that more accurately mirror these transporter expression patterns may offer more dependable insights when conducting transport studies on IBD patients.

**Table 2:** In vitro culture models for IBD

*Cell lines*				
Cell types	Pros	Cons	Inflammatory stimuli	Ref
Caco-2	▪ Barrier forming ▪ Reproducibility ▪ Cheap ▪ Ease to handle	▪ Only epithelial cells ▪ Limited transporter expression ▪ Carcinoma-derived ▪ Limited differentiation	DSS	(Araki et al. 2006; Toutounji et al. 2020)
			TNF-α	(Liang et al. 2020)
			IL-1β	(Maria-Ferreira et al. 2018)
			LPS	(Wang et al. 2023)

*Co-cultures*				
Cell types	Pros	Cons	Inflammatory stimuli	Ref
Caco-2/HT29-MTX	▪ Barrier forming ▪ Reproducibility ▪ Cheap ▪ Ease to handle ▪ Mucus production	▪ Only epithelial cells ▪ Limited transporter expression ▪ Carcinoma-derived	IL-1β TNF-α Hypoxia	(Dosh et al. 2019)
Caco-2/THP-1	▪ Barrier forming ▪ Immune response ▪ Cheap	▪ Limited transporter expression ▪ Carcinoma-derived ▪ Limited differentiation	IFN-γ + LPS	(Kämpfer et al. 2017)
Caco-2/HT29-MTX/THP-1	▪ Barrier forming ▪ Immune response ▪ Mucus production	▪ Limited transporter expression ▪ Carcinoma-derived ▪ Increased complexity	IFN-γ + LPS	(Kämpfer et al. 2022)
			LPS	(Marescotti et al. 2021)
Caco-2/THP-1/MUTZ-3	▪ Barrier forming ▪ Immune response ▪ Both monocyte- and dendritic cell-like cell types	▪ Limited transporter expression ▪ Carcinoma-derived ▪ Increased complexity ▪ Limited differentiation	LPS	(Paul et al. 2023)
			IL-1β	(Susewind et al. 2016)
Caco-2/PBMC-derived macrophages	▪ Barrier forming ▪ Immune response	▪ Limited transporter expression ▪ Increased complexity ▪ Limited differentiation	LPS	(Schnur et al. 2022)

*Stem cell cultures*				
Cell types	Pros	Cons	Inflammatory stimuli	Ref
iPSC-derived HIO	▪ Differentiated epithelial layer ▪ Commercially available ▪ IBD patient-derived iPSCs	▪ Spherical ▪ Fetal-like phenotype ▪ No immunologic component ▪ Donor variability ▪ High-maintenance ▪ Costly	IFN-γ	(Workman et al. 2020)
			NA	(Sarvestani et al. 2021)
			TGF-β	(Estrada et al. 2022)
ASC-derived HIO	▪ Differentiated epithelial layer ▪ IBD patient-derived HIO with inflammatory phenotype ▪ Adult phenotype	▪ Spherical ▪ No immunological component ▪ Access to mammalian tissue required ▪ Donor variability ▪ Costly	TNF-α	(Lee et al. 2021)
			TNF-α + IL-1β + IL6	(d’Aldebert et al. 2020)
IBD patient-derived HIO monolayers	▪ Barrier forming ▪ IBD patient-derived iPSCs	▪ Spherical ▪ Fetal-like phenotype ▪ No immunologic component ▪ Genetic variability ▪ High-maintenance ▪ Costly	IFN-γ + TNF-α + IL-1α	(Jelinsky et al. 2023)

Since the Caco-2 model mainly represents enterocytes, co-culturing with other cell types can provide a more physiologically relevant model. Co-culturing Caco-2 cells with HT29-MTX cells that resemble a goblet cell-like phenotype provides a model with mucus as an additional barrier against pathogens or toxicants (Hoffmann et al. 2021). Tri-culture models that additionally include THP-1 derived macrophages as immune cells have been used to emulate inflammatory conditions on a cellular level like observed in IBD (Gijzen et al. 2020). Caco-2/THP-1 co-cultures can be stimulated with IFN-γ and LPS to induce inflammation followed by barrier disruption, cytokine release, and cytotoxicity (Kämpfer et al. 2017).

While immortalized cell line (co)culture models have proven to be most valuable tools in intestinal research, they lack cell diversity, tissue architecture, and overall biologic complexity as seen in vivo. Therefore, human stem cell-derived in vitro models are increasingly being explored as models for IBD and to study the interaction with chemicals.

#### Stem cell-derived intestinal models

Several types of stem cell-derived models are being developed. Commonly, stem cells are grown in vitro as organoids. Organoids are three-dimensional structures that self-organize through cell–cell and cell–matrix interactions to recapitulate intestinal epithelial aspects in vitro (Marsee et al. 2021). Co-culturing organoids with immune cells, stromal cells, endothelial cells or a microbiome renders them complex in vitro models that can emulate the intestinal microenvironment (Puschhof et al. 2021; Hentschel et al. 2021). The spherical nature of organoids limits their applicability for apical exposure studies, therefore 2D stem-cell derived cell layers that self-organize, self-renew and self-polarize are currently being developed (Wang et al. 2017; Grouls et al. 2022). Stem cell-based culture models are derived from two primary sources of stem cells. First, adult stem cells can be directly derived from human tissue, including from tissue from IBD patients (Dotti et al. 2022). Human intestinal organoids derived from CD patients were found to have an impaired epithelial regeneration upon TNF-α stimulation compared to healthy controls (Lee et al. 2021), illustrating the effectiveness of such a model for exposure studies. Interestingly, a cocktail of IL-1β, IL6, and TNF-α was able to reproduce this inflammatory phenotype in healthy control organoids (d’Aldebert et al. 2020). The second main type of stem cells are induced pluripotent stem cells (iPSCs). These cells are obtained by reprogramming somatic cells into pluripotent cells (Chen et al. 2014) that can be further differentiated into intestinal epithelial cells (Shafa et al. 2018). Exposure of such iPSC-derived intestinal cell models to interferon-γ resulted in tight junction disruption and an increase in the expression of IBD-associated genes (Workman et al. 2020). iPSC-derived organoids from very early onset IBD patients could be used to model fibrotic responses in vitro in response to TGF-β (Estrada et al. 2022). Furthermore, iPSC-derived organoids of UC patients were found to recapitulate histological and functional features of in vivo colitis (Sarvestani et al. 2021). iPSCs can also be developed into cell layers, but these layers show a more fetal-like phenotype compared to adult stem cell-derived models (Negoro et al. 2018).

#### Advanced tissue and cell culturing platforms

Microphysiological systems, such as organs-on-chips, hollow-fiber membranes, and microfluidic chambers are emerging techniques in the field of organ modelling (Nitsche et al. 2022). These in vitro systems can be used to mimic the architecture, circulation, and mechanical stress of the intestine (Amirabadi et al. 2022). Microfluidic devices allow for a tight control of the tissue microenvironment, by controlling for example oxygen levels (Richardson et al. 2020), by emulating intestinal luminal conditions and incorporating IBD patient-derived microbiota (Donkers et al. 2024), or by the incorporation of scaffolds to emulate the extracellular matrix (Cherwin et al. 2023). Intestinal tissues from IBD patients cultured in such systems remain to express inflammatory markers, as measured by calprotectin release (Dawson et al. 2016).

Microphysiologic culture systems have been used to culture intestinal cell lines and intestinal stem cells (Kasendra et al. 2018; Ingber 2022; Shin and Kim 2022) in an attempt to recreate more in vivo-like intestinal phenotypes. The incorporation of Caco-2 and endothelial cell co-cultures in such a culture system allowed the recreation of IBD-like intestinal tissues (Tataru et al. 2023). Furthermore, the flow of cell culture media prevented bacterial overgrowth as observed in statit culturing methods and therefore allows to co-culture intestinal microorganisms with human cells (Shin and Kim 2018). In addition, stem cell-derived models have been included in micro-physiologic culture systems using adult stem cells from IBD patients (Shin et al. 2020). Interestingly, micro-physiologic culture systems allow the culture of different tissues in separate compartments while still allowing communication of cells and signaling molecules, which increases functionality of the cell models. Using a gut-liver co-culture model, the modulatory role of microbial short-chain fatty acids in IBD related intestinal inflammation was studied (Trapecar et al. 2020). While OoC platforms hold great potential for next-generation risk assessment of chemicals, overcoming certain challenges is essential before these platforms can effectively contribute to the evaluation of pharmacokinetic–pharmacodynamic parameters.

### Future directions of toxicological research using advanced cell models for IBD

The toxicological safety assessment of chemicals traditionally focuses on protecting the general population, which does not necessarily incorporate individuals with an impaired intestinal barrier. From a toxicologists perspective, the increased prevalence of IBD raises concerns on a potentially increased bioavailability of chemicals and drugs. As discussed, altered intestinal tissue functionality during disease exacerbation can affect the toxicokinetic and toxicodynamic behavior of drugs, foodborne chemicals and particulate matter. Given the increasing prevalence of IBD this increases the relevance of considering IBD patients as a vulnerable population within the toxicological risk assessment of chemicals. In addition, evidence is accumulating that chemical and particulate matter exposure via food and drinking water can attenuate intestinal inflammation in IBD patients as well as (vulnerable) healthy individuals.

While several IBD animal models are available to study the consequences of chemical and particle exposure on the disease pathophysiology, these rodent models can be limited in their ability to mechanistically study the interactions, and neither can they be used to study the underlying causes for disease initiation (and the role of environmental factors on this initiation). Other animal models can be considered, such as the zebrafish, which can prove a valuable tool due to their low maintenance costs, fecundity, genetic similarity to humans, ease of gene-editing, and optical transparency at the embryonic level (Hanyang et al. 2017; Choi et al. 2021). However, non-animal models offer greater advantages and can exploit different routes. Ex vivo intestinal tissue approaches allow to study the consequences of chemical exposure on the complex tissue microenvironment, but require easy access to human (surgery) material and its use is limited given the short life-span of the tissue segments.

Advanced in vitro models, ranging from co-culture models with intestinal cell lines to iPSCs and adult stem cells, are good candidate models to study interaction of chemicals on intermediate mechanistic steps, so-called key events, in the pathophysiology of IBD. Obviously, this is currently being explored within toxicological sciences in the adverse outcome pathway (AOP) approach, which was launched a decade ago to structure the integration of ex vivo and in vitro models in toxicological risk assessment (Vinken 2013). Both ex vivo models and stem cell-derived models allow to use patient-derived material, which has great addition benefits compared to cell line models (and animal models). The application and advantages of using stem cell models in biomedical sciences is apparent, as it allows a personalized approach into the therapeutic potential of drugs and chemical vulnerability.

## Conclusions

Risk assessment of foodborne contaminants and drugs is primarily performed to protect healthy individuals from adverse health outcomes. As patients suffering from IBD show an impaired intestinal barrier, as well as altered transport and defence mechanisms in the intestinal epithelium, the outcome of these risk assessments might not hold true for this sub-group of the population. As the prevalence of IBD is strongly increasing in the western world, we recommend to consider individuals with prevalent intestinal inflammation in the risk assessment process of food contaminants and orally applied drugs. Currently available experimental models of IBD still carry disadvantages regarding costs, complexity or disease onset, but might prove to be valuable tools in future.

## Data Availability

Not applicable for this manuscript.

## Abbreviations

IBD: Inflammatory bowel disease
CD: Crohn’s disease
UC: Ulcerative colitis
SNPs: Single nucleotide polymorphisms
IL: Interleukins
TNF-α: Tumour necrosis factor-alpha
IOIBD: International Organization for the Study of Inflammatory Bowel Disease
DON: Deoxynivalenol
ENM: Engineered nanomaterials
TiO2: Titanium dioxide
SiO2: Silica
bw: Body weight
NLRP3: NOD LRR- and pyrin domain-containing protein 3
CDAI: Crohn's disease activity index (CDAI)
CPF: Chlorpyrifos
BPA: Bisphenol A
FDA: Food and Drug Administration
EFSA: European Food Safety Authority
CMC: Carboxymethylcellulose
P80: Polysorbate 80
5-HT: 5-Hydroxytryptamine
ENT: Equilibrative nucleoside transporter
CNT: Concentrative nucleoside transporter
OATP: Organic anion-transporting polypeptide
MRP: Multidrug resistance protein
ASBT: Apical sodium-dependent bile acid transporter
OST: Organic solute transporter
OCTN: Novel organic cation transporter
MCT: Monocarboxylate transporter
P-gp: P-glycoprotein
BCRP: Breast cancer resistance protein
PPI: Protein pump inhibitors
NSAIDS: Nonsteroidal anti-inflammatory drugs
COX: Cyclooxygenase
PMN: Polymorphonucleocyte
SSRIs: Selective serotonin reuptake inhibitors
SERT: Serotonin transporter
AUC: Area under the plasma concentration–time curve
DSS: Dextran sulfate sodium
TNBS: Trinitrobenzene sulfonic acid
Th: T-helper
LPS: Lipopolysaccharide
TLR4: Toll-like receptor 4
iPSCs: Induced pluripotent stem cells
AOP: Adverse outcome pathway
TJ: Tight junction
